# From M. Hartwig, M. D.

**Published:** 1885-10

**Authors:** M. Hartwig

**Affiliations:** Buffalo, N. Y.


GENTS—Among your selections, September, 1885, you cite two cases of sunstroke successfully treated with subcutaneous injections of antipyrin. I am sorry to report a negative result with exactly the same treatment. During the short heated term we had this year, I was called to see a very robust man in frightful opisthotonic convulsions, which had started two hours before my arrival, while the man was working in a brick-yard. Coma, enormous heat of the body, perhaps 108. The very short intervals between the tetanic spells prevented measuring the temperature at that time. All the same, I do not doubt the correctness, because I know I hardly ever fail to guess correctly within the limit of one degree. Internal remedial administration being out of question, I proceeded just as Westbrook has in his cases. I had cold water affused for one-half hour over head and chest, injected subcutaneously grs. xx. antipryrin with gr. 1-6 morphine, and drew from the cubital vein one pint of blood. Ordering ʒss chloral, ʒss kalii brom. and grs. xx. chin. sulp. per clysma. I left at 12 o'clock. Between 5 and 6 in the afternoon, temperature in the rectum was 102. Tetanic convulsions less frequent, coma. The people objecting to further handling of the "dying" man as useless, I left, in doubt whether the rectal injection was correctly executed. The man died the same night. Whether a larger dose of antipyrin would have been more successful, I doubt, because the temperature had diminished sufficiently not to form a dangerous momentum in itself. The injury to the nerve cells sustained by the preceding heat must have been sufficient to kill. Neither your report nor one I saw in the *Medical Digest* gives the time of Dr. Westbrook's arrival. Notwithstanding my negative result, I would repeat the proceeding in another case; or if antipyrin should not be at hand, I would inject subcutaneously chinium muriaticum carbamidatum, which dissolves in the same amount of water, weight for weight. This drug saved a case of endometritis septicæ with coma and very high temperature in my hands, and I doubt not would work like antipyrin in sunstroke. It is used altogether too seldom.

Respectfully yours,

Dr. M. Hartwig.

Buffalo, N. Y., September 17, 1885.

